# Goldenhar Syndrome: A Case Report with Review

**DOI:** 10.5005/jp-journals-10005-1377

**Published:** 2016-09-27

**Authors:** Mridula Goswami, Urvashi Bhushan, Babita Jangra

**Affiliations:** 1 Professor and Head, Department of Pedodontics and Preventive Dentistry Maulana Azad Institute of Dental Sciences, Delhi, India; 2 Postgraduate Student, Department of Pedodontics and Preventive Dentistry Maulana Azad Institute of Dental Sciences, Delhi, India; 3 Postgraduate Student, Department of Pedodontics and Preventive Dentistry Maulana Azad Institute of Dental Sciences, Delhi, India

**Keywords:** Early diagnosis, Goldenhar syndrome, Multidis-ciplinary approach.

## Abstract

Goldenhar syndrome is a rare condition which is characterized by a multitude of anomalies involving craniofacial structures, vertebrae, internal organs and usually occurs unilaterally. The etiology of this syndrome is unclear since it varies genetically and is linked to a plethora of reasons. Herein, we report a case of Goldenhar syndrome with hemifacial microsomia and microtia along with systemic involvement which was clinically and radio-graphically assessed. Many classical signs of the syndrome were present in the patient along with few rare ones. The various aspects of this rare disease have been discussed with emphasis on timely diagnosis and multidisciplinary approach to manage it.

**How to cite this article:**

Goswami M, Bhushan U, Jangra B. Goldenhar Syndrome: A Case Report with Review. Int J Clin Pediatr Dent 2016;9(3):278-280.

## INTRODUCTION

Goldenhar syndrome, also known as oculo-auriculo-vertebral syndrome (OAVS), is a rare congenital condition arising from defects in the first and second brachial arches. It was first described by Dr. Maurice Goldenhar in 1952.^1^ The incidence of Goldenhar syndrome has been reported to be 1:35,000-1:56,000 with a male to female ratio of 3:2.^2^ Etiology of this condition is not yet fully established. Abnormalities of chromosomes, neural crest cells, environmental factors during pregnancy like ingestion of drugs, such as cocaine, thalidomide, retinoic acid, intake of alcohol by the mother were also related to the development of the disease. Maternal diabetes has also been suggested as an etiologic factor.^3^ Clinically, the patient may present with a variety of features ranging from facial abnormalities, ear abnormalities, eye abnormalities, vertebral defects, and congenital heart problems.

In this article, we report a case of Goldenhar syndrome along with discussion on clinical features, importance of early diagnosis, and interdisciplinary approach to manage it.

## CASE REPORT

**Fig. 1:**
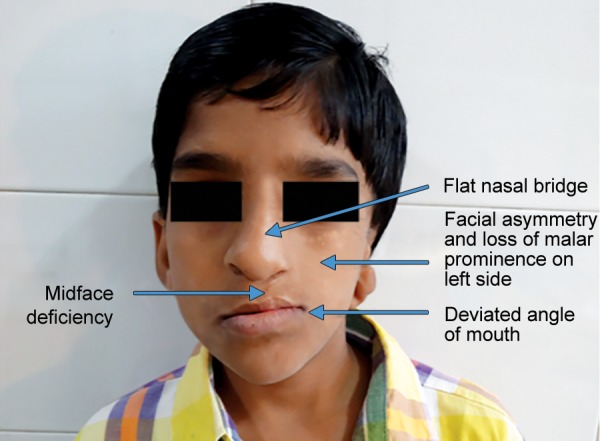
Extraoral picture of the patient

**Fig. 2:**
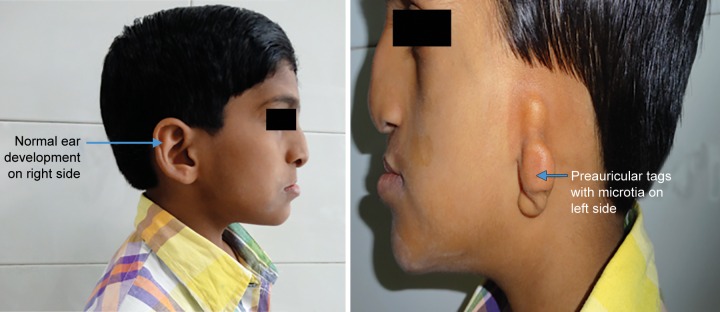
Normal morphology of right ear and deformed left ear with preauricular tags and microtia

**Fig. 3:**
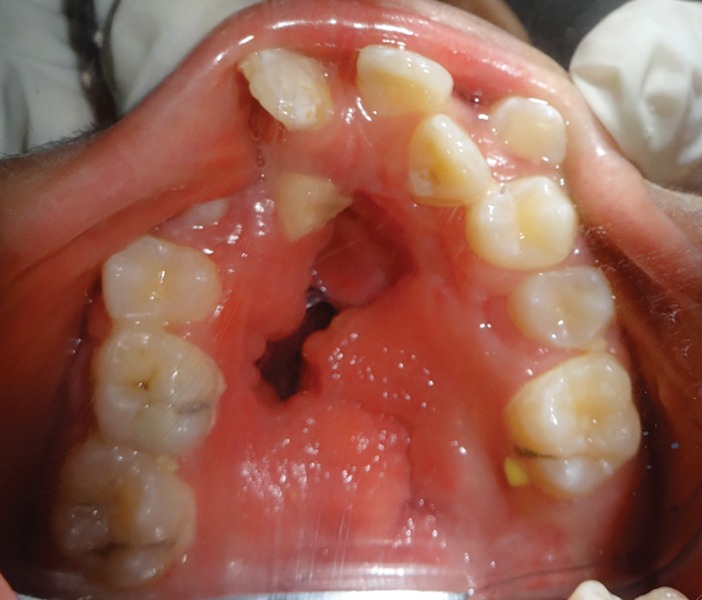
Cleft palate with constricted maxillary arch

**Fig. 4:**
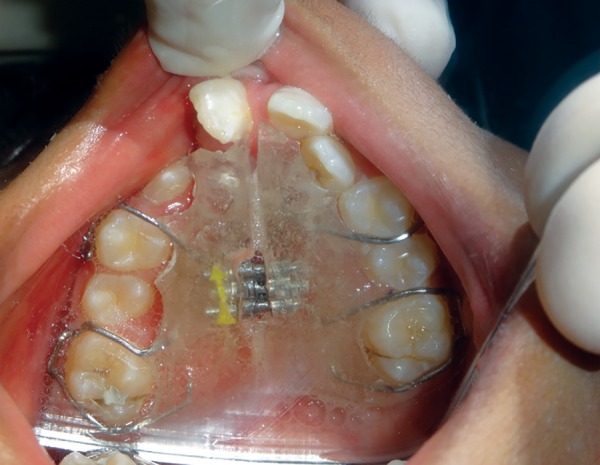
Obturator with midline expansion screw

**Fig. 5:**
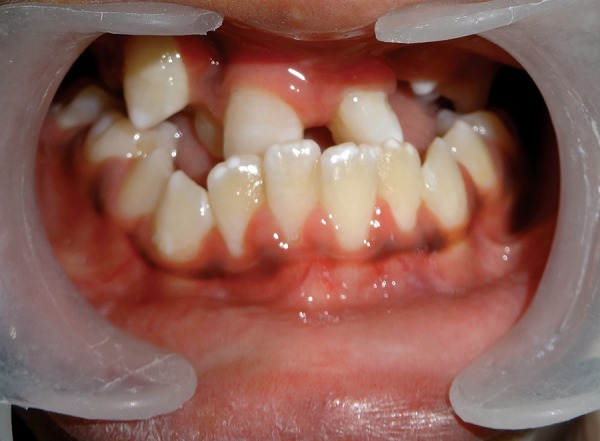
Severe class III malocclusion

A 10-year-old patient reported to the Department of Pedodontics and Preventive Dentistry, Maulana Azad Institute of Dental Sciences, New Delhi, with the chief complaint of difficulty in eating and drinking. There was no history of trauma to head and neck region or maternal exposure to teratogenic agents. No signs of mental retardation or impairment of cognitive function were seen. There was history of acyanotic congenital heart disease with congestive heart failure, ventricular septal defect, and repeated episodes of pneumonia. On extraoral examination, facial asymmetry was present with deviation of angle of mouth toward the left side (Fig. 1). There was flattening of left side of face with loss of left malar prominence. Facial profile was concave due to midface deficiency. Microtia along with preauricular tags was present on the left side (Fig. 2). Intraoral examination revealed cleft palate with constricted maxillary arch (Fig. 3). Class III malocclusion was present with crowding in maxillary and mandibu-lar dentition. Orthopantomograph showed hypoplastic condyle, ramus, and body of mandible on left side. An obturator was fabricated which incorporated midline expansion screw for expanding the narrow maxillary arch (Fig. 4). An extensive orthodontic therapy was planned for correcting class III malocclusion (Fig. 5) and probable surgical intervention after growth cessation in collaboration with orthodontists as well as oral and maxillofacial surgeons, in the future. Patient has been kept on regular follow-up and observation for other possible malfunctions in craniofacial region or systemic organs.

## DISCUSSION

Goldenhar syndrome is characterized by a triad of accessory tragi, mandibular hypoplasia, and ocular dermoids. It is an extremely rare, inherited syndrome with unclear etiology and occurs sporadically. In some cases, positive family histories have been present that have suggested autosomal dominant or recessive inheritance. Some researchers suggested multifactorial mode of inheritance due to interaction of several genes, possibly in combination with environmental factors. Abnormal vascular supply of embryo, disrupted mesodermal migration can lead to defective formation of brachial arches and vertebral system.^4^ Mounoud et al^5^ reported a case where mother had history of vitamin A intoxication, which could have led to this disease. This teratogen produces ill effects on neural crest cell formations, which are essential for the formation of pharyngeal arches.^6^

The characteristic combination of external ear anomalies and ipsilateral facial underdevelopment is the hallmark of Goldenhar syndrome as seen in our case.^7^ The condition is mostly unilateral in occurrence in 85% cases, with the right side more frequently affected than the left with a ratio of 3:2.^8^ In our case, unilateral facial involvement of left side was seen which made it a more rare category. Mandibular hypoplasia is usually seen in patients, though in our case maxillary deficiency was seen with class III malocclusion. Systemic involvement in Goldenhar syndrome can vary widely too. Among cardiovascular anomalies, tetralogy of Fallot and ventricular septal defects are most commonly associated with OAVS.^9^ Cleft lip and palate, macrostomia, micrognathia, webbing of the neck, a short neck, tracheoesophageal fistulas, and abnormalities of sternocleidomastoid muscle may be associated. Cleft lip/palate was also present in our patient. Although diagnosis is mainly clinical, radiographic investigations help to support the clinical diagnosis. Prenatal diagnosis is possible with considerable accuracy with ultrasound which may detect obvious defects. Since no specific genes have been linked to this syndrome, prenatal deoxyribonucleic acid testing cannot be used to diagnose the condition. The differential diagnosis is broad, necessitating it to be distinguished from other syndromes like Treacher Collins syndrome (TCS), Nager syndrome and Townes-Brocks syndrome. In Goldenhar syndrome, usually unilateral facial involvement is seen leading to facial asymmetry, while in TCS, involvement is frequently bilateral. Mutation of TCOF1 gene on human chromosome 5q31-34 is specifically linked to TCS and helps in the final diagnosis.

Treatment of disease varies with age and according to systemic associations. In patients with mandibular hypoplasia, reconstruction can be done with rib grafts, and underdeveloped maxilla can be lengthened by bone distraction device. Structural anomalies of the eyes and ears can be corrected by plastic surgery. Surgical corrections for cleft lip and palate can be done, followed by an orthodontic correction after jaw growth completion. Patient may suffer from severe obstructive sleep apnea caused by airway abnormalities and jaw growth deficiency, which leads to restrictive diet and malnutrition. Maintenance of oral hygiene is difficult in Goldenhar syndrome due to malocclusion, limited mouth opening, and intellectual impairment, making them prone to dental caries and gingivitis. So, management requires a multidisciplinary approach to cater to the multitude of anomalies in the affected child. Prognosis is guarded in cases with systemic involvement, but is good in otherwise uncomplicated cases without any systemic associations.

## CONCLUSION

Role of a pedodontist is significant to ensure optimum oral health care for such syndromic patients from birth till adolescence since often they have complex unmet dental needs. Pediatric dentists and pediatricians should work in collaboration with sound referral services for prompt treatment of the affected children. Efforts should be made by the medical and dental community to diagnose and manage this condition at the earliest, lessening the emotional, physical, and financial burden of living in these special children.

## References

[1] Goldenhar M (1952). Associations malformatives de l’oeil et l’oreille, en particulier le syndrome dermoide epibulbaire-appendices auriculaires-fistula auris congenita et ses relations avec la dysostose mandibulo-faciale.. J Genet Hum.

[2] Ashokan CS, Sreenivasan A, Saraswathy GK (2014). Goldenhar syndrome―review with case series.. J Clin Diagn Res.

[3] Araneta MR, Moore CA, Onley RS, Edmonds LD, Karcher JA, McDonough C, Hiliopoulos KM, Schlangen KM, Gray GC (1997). Goldenhar syndrome among infants born in military hospital to Gulf war veterans.. Teratology.

[4] Kulkarni VV, Shah MD, Parikh AA (1985). Goldenhar syndrome: a case report.. J Postgrad Med.

[5] Mounoud RL, Klein D, Weber F (1975). A case of Goldenhar syndrome: acute vitamin A intoxication in the mother during pregnancy.. J Genet Hum.

[6] Mathur MV, Mishra Lt Col RK (2000). Golden-har syndrome.. MJAFI.

[7] Vinay C, Reddy RS, Uloopi KS, Madhuri V, Sekhar RC (2009). Craniofacial features in Goldenhar syndrome.. J Indian Soc Pedod Prev Dent.

[8] Kokavec R (2006). Goldenhar syndrome with various clinical manifestations.. Cleft Palate Craniofac J.

[9] Werler MM, Starr JR, Cloonan YK, Speltz ML (2009). Hemifacial microsomia: from gestation to childhood.. J Craniofac Surg.

